# Effect of Anconeus Muscle Blocking on Elbow Kinematics: Electromyographic, Inertial Sensors and Finite Element Study

**DOI:** 10.1007/s10439-016-1715-2

**Published:** 2016-08-29

**Authors:** Israel Miguel-Andres, Teresa Alonso-Rasgado, Alan Walmsley, Adam C. Watts

**Affiliations:** 1 Bioengineering Research Group, School of Materials, The University of Manchester, Manchester, M13 9PL UK; 2 Wrightington Hospital, Wigan and Leigh NHS Foundation Trust, Lancashire, UK

**Keywords:** Lidocaine, Weak extensor, Flexion–extension, Pronation–supination, Net torque, Power

## Abstract

The specific contribution of the anconeus muscle to elbow function is still uncertain. This study aimed to investigate the effect on elbow kinematics and kinetics of blocking anconeus using lidocaine. Ten healthy volunteers performed experimental trials involving flexion–extension and supination–pronation movements in horizontal and sagittal planes. Inertial sensors and surface electromyography were used to record elbow kinematics and kinetics and electrical activity from the anconeus, biceps and triceps brachii before and after blocking anconeus. Moreover, a finite element model of the elbow was created to further investigate the contribution of anconeus to elbow kinematics. The electrical activity results from the trials before blocking clearly indicated that activity of anconeus was increased during extension, suggesting that it behaves as an extensor. However, blocking anconeus had no effect on the elbow kinematics and kinetics, including the angular velocity, net torque and power of the joint. The electrical activity of the biceps and triceps brachii did not alter significantly following anconeus blocking. These results suggest that anconeus is a weak extensor, and the relative small contribution of anconeus to extension before blocking was compensated by triceps brachii. The finite element results indicated that anconeus does not contribute significantly to elbow kinematics.

## Introduction

The anconeus muscle has been implicated in common elbow conditions such as lateral epicondylosis and posterolateral rotatory instability (PLRI).^10^ PLRI is the most common instability of the elbow. The anconeus muscle is currently used in clinical practice in the rehabilitation of patients with this condition as it is thought to be an important secondary stabiliser of the elbow.^15^,^22^

Patients are trained in anconeus activation and strengthening programmes are initiated. This function may be compromised if iatrogenic denervation occurs. The anconeus may be denervated during surgical approaches to the elbow such as when performing elbow arthroscopy, or an olecranon osteotomy.^18^,^31^,^36^,^37^,^50^,^51^ While surgical approaches have been described that will preserve anconeus function they increase surgical time and are often avoided because the anconeus may be perceived to be unimportant.^38^ Whilst some studies have reported that patients have experienced no deficiencies in elbow function after anconeus sacrificing surgery,^31^,^50^ these claims have been questioned and a more recent study has suggested that there may be consequences for elbow function and stability of removing anconeus.^5^

If it is shown that anconeus is important to normal elbow kinematics then surgeons would be encouraged to employ techniques that preserve the muscle and the nerve supply to it.

The specific function and contribution of the anconeus muscle to elbow kinematics is yet to be clearly established. Electromyographic studies have found that the electrical activity of the anconeus is greater in extension,^28^^–^ 30,41,44,53 suggesting that it is an elbow extensor muscle. Furthermore, it has been proposed that the anconeus could abduct the ulna during pronation.^13^ This idea was later supported by Ray *et al*.^46^ and Gleason *et al*.^20^ Other studies have observed that the anconeus is active during resisted pronation–supination movement, implying that it could contribute to elbow stability.^24^,^41^,^53^ In addition, several studies have suggested that the strong adherence between the lateral joint capsule and the anconeus makes the latter an active stabiliser of the elbow.^3^,^35^,^39^

Recent anatomical studies have been undertaken to clarify the function of anconeus. For example, Coriolano *et al*.^12^ found that the muscle fibres of anconeus were penniform, a formation proficient for force production. In another anatomical and biomechanical study, it was observed that anconeus behaves as an extensor muscle and provides posterolateral stability of the elbow.^42^

Although it has been argued that anconeus is an accessory muscle at the elbow, some researchers have suggested that the muscle could contribute up to 15% of the extension moment during isometric contractions.^56^ In addition, it has been suggested that anconeus plays an important role when torque values are low.^29^,^30^ However, a re-examination of these results indicates that anconeus was active tonically at torques below 39 Nm and exhibited approximately constant activity at all elbow angles at a constant torque of 6 Nm. In addition, anconeus activity was observed to increase markedly with increasing angular velocity with maximum activation occurring concurrently with maximum angular velocity. The close correspondence with maximum angular velocity appears to indicate that anconeus contributes to the centripetal force required to maintain joint integrity.

A potential reason as to why research to date has failed to elucidate anconeus function was put forward by Capdarest-Arest *et al.* 8 who suggested there is evidence to argue that the anconeus muscle has a primary function as a stabilizer in human infants during the period when infants crawl but that as they grow and develop, the anconeus then takes on a more accessory-type role.

The current study aims to elucidate the role of anconeus in order to determine its importance to normal elbow kinematics and in doing so to help inform the debate as to whether surgeons should be encouraged to employ techniques that preserve the muscle and the nerve supply to it during surgery or could potentially consider its sacrifice without a risk to elbow function. In this study the role of anconeus was investigated by determining the effect of blocking the action of anconeus muscle on elbow kinematics and kinetics, and the activation of biceps and triceps brachii, using experimental trials and computational modelling.

## Materials and Methods

In order to clarify the contribution of the anconeus muscle to the motion of the elbow, the electrical activity of anconeus, biceps and triceps were recorded both before and after the anconeus muscle was blocked with lidocaine. Measurements were taken using electromyography (EMG) and motion tracking devices (3D inertial sensors) during the performance of three flexion–extension movements and supination–pronation.

### Subjects

Ten right-handed volunteers, five males and five females, mean age 29.3 ± 2.21 years and mean body mass 66.80 ± 9.56 kg, with no history of neuromuscular or musculoskeletal disease took part in this investigation. All trials were carried out using the dominant hand of the participant. The participants were informed about the procedures before the tests and the protocol was approved by the ethics committee of the University of Manchester (approval number 11335).

#### Subject Preparation

First, the skin on the belly of anconeus, biceps and triceps brachii of each participant was cleaned with 70% isopropyl alcohol and then shaved before being cleaned for a second time.

For the trials where the anconeus was blocked, the skin was prepared with an alcoholic chlorhexidine solution, then a trained orthopaedic surgeon injected 10 ml of plain lidocaine through a 25 gauge hypodermic needle into the soft tissue at a point midway between the tip of the olecranon and the lateral epicondyle of humerus. The injection was performed with the elbow flexed approximately 90°. At least 10 min were allowed for the local anaesthetic to work before the experimental protocol was initiated.

### Equipment

#### Myoelectric Activity: Electromyography (EMG)

A BTS pocket EMG Electromyography system (BTS Bioengineering, Milan, Italy) was used to record the electrical activity of the anconeus, biceps and triceps brachii muscles. Bipolar paediatric electrodes (Ag/AgCl) were used at a sampling frequency of 2000 Hz to obtain the required data from these muscles. Electrodes were positioned on the belly of the anconeus, biceps and triceps brachii muscles and on the styloid process of the ulna at the wrist. The electrode placed on the ulna was used for ground/reference purposes.

#### Kinematics and Kinetics: Inertial Measurement Units (IMUs)

To obtain the kinematic data for the elbow of each participant, 3D wireless inertial measurement units (Xsens Technologies, Enschede Netherlands) were used. Before the trial began, the sensors were calibrated by placing them on a flat surface and then executing a heading reset. This was to ensure that the gravitational acceleration vector and the global Z axis remained vertical.

**Figure 1:**
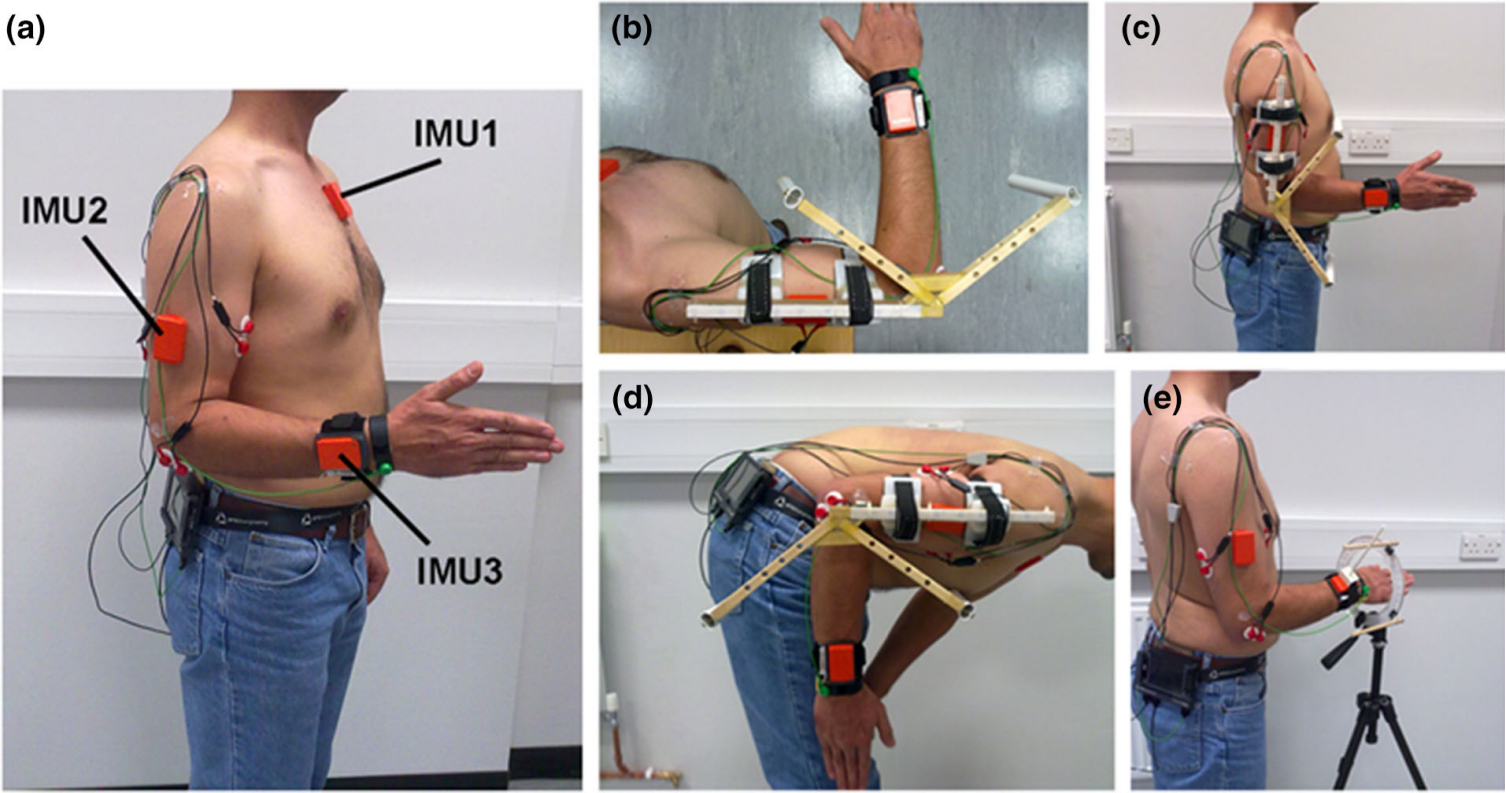
(a) Participant preparation. Surface electrodes and inertial measurement units (IMUs) were placed on the upper limb and thorax. Elbow movements: (b) flexion–extension with shoulder abducted 90°; (c) flexion–extension in the sagittal plane with the elbow close to the trunk; (d) flexion–extension with the spine bent forward 90° and (e) Supination-pronation with the elbow flexed 90°.

Data were acquired at a sampling rate of 75 Hz. Three inertial measurement units (IMUs) were employed; the first sensor (IMU1) was attached below the external notch of the thorax. The second (IMU2) and third (IMU3) sensors were placed on the lateral side of the arm and on the posterior side of the wrist respectively, as shown in Fig. 1a. Surface EMG and IMU data collection was synchronised using an external trigger during the trials, enabling the electrical muscle activity and elbow kinematics to be recorded simultaneously.

### Motions

The participant performed flexion–extension and supination–pronation movements in both transverse and sagittal planes, as shown in Fig. 1b–e. The motions were performed in four different postures enabling the function of the anconeus to be investigated. The motions considered were as follows:

- Flexion–extension in a horizontal plane with the shoulder abducted 90°
- Flexion–extension in a sagittal plane while standing with the elbow close to the trunk
- Flexion–extension in a sagittal plane with the spine bent forward 90° and the upper arm aligned horizontally and parallel to the ground
- Supination–pronation with the elbow flexed 90°

The active range of motion for flexion–extension was constrained to 90° by a custom-made frame constructed out of wood. The frame was attached to the lateral side of the arm. Supination–pronation motions were constrained to the same range by a custom-made Perspex ring.

### Procedure

The EMG and IMU measurements for each participant were repeated before and after the anconeus muscle was blocked. Before the tests began a static trial was undertaken by each participant in order to determine the level of electrical activity resulting from cross talk from adjacent muscles, noise and soft tissue artefact.^16^ The EMG from all muscles was measured over a period of 20 s with the subject standing still in the fundamental position. The mean magnitude of the background EMG from the static trials was approximately 2% MVC.

#### Before Blocking the Anconeus Muscle

Once the skin of the participant had been prepared, the EMG electrodes (Ag/AgCl) placed, and the three IMUs attached, the participant was asked to perform dynamic trials involving flexion–extension and supination–pronation motion of the dominant arm while EMG and IMU data were recorded.

The flexion–extension movements were performed with the forearm in neutral rotation. Each trial consisted of 5 cycles performed over a period of approximately 28 s paced using a metronome. Each cycle was paced by 4 beeps 1 s apart; the first and second beeps were to start and stop flexion and the third and fourth beeps were to start and stop the extension motion. The flexion–extension motion was constrained to 90° by a custom-made wooden frame to ensure (Fig. 1b–1d) that all participants reliably performed the same range of motion during the trials. The correct posture of the elbow and trunk during the tests was monitored by visualisation of the orientation of the arm and thorax sensors in real time.

For the supination–pronation motion, the participants performed three trials with the elbow flexed 90°, pointing at a fixed reference with the middle finger.^20^ The motion was constrained to 90° using a Perspex ring, as shown in Fig. 1e. The movements were paced using the metronome at the same frequency used for the flexion–extension movements.

#### After Blocking the Anconeus Muscle

Once the participants had undertaken the first set of dynamic trials, the anconeus muscle was blocked using 10 ml of plain lidocaine. Ten minutes were allowed for the local anaesthetic to work before the second set of dynamic trials was started. To ensure that the anconeus muscle was effectively blocked, the electrical activity of the muscle was measured when the participants flexed and extended the elbow three times with the spine bent forward 90° because, in this position, anconeus was clearly active before applying the anaesthesia. Once it was determined that anconeus was effectively blocked, the second set of dynamic trials was initiated, which entailed participants undertaking a second set of flexion–extension and pronation–supination movements following the same procedure used for the first set of trials completed prior to blocking anconeus.

### Data Processing

The EMG data from the dynamic trials were normalised to 100% MVC to enable comparison between conditions. Participants were asked to perform isometric maximum voluntary contractions (MVC) in order to detect the maximum electrical activity of the muscles. Each participant performed three isometric maximum voluntary contractions (MVC) of 6 s duration for each muscle. The participants were encouraged verbally during the tests to get the maximum muscle activity. Two minutes of rest between each MVC test were given to minimise muscle fatigue.

The root mean square (RMS) amplitude of every MVC test was calculated within a window of 1.5 s and then the maximum RMS amplitude of each muscle was used to normalise the EMG from the dynamic trials in each volunteer. The data were filtered with a 2-pole Butterworth band pass filter with cut-off frequencies of 5 and 600 Hz in order to retain as much as possible of the electrical activity data.^32^ Subsequently, the muscle activity data were rectified and filtered with a 2-pole zero-lag Butterworth low pass filter with a cut-off frequency of 6 Hz.^48^ The electrical activity data from the 15 cycles from the 3 trials undertaken by each participant for each motion were averaged and time normalised from zero to 100% of the movement time. The normalised and averaged data from all participants were combined to give an overall average for all the participants.

The raw data from the IMUs were filtered with a 2-pole zero lag Butterworth low pass filter with a cut-off frequency of 2 Hz. This cut-off frequency was obtained from a frequency spectrum analysis of the elbow angular velocity.^55^ The data processing was performed in MATLAB version 8.2.0.701. The filtered IMU data from the 15 cycles from the 3 trials undertaken by each participant for each motion were also averaged and time normalised.

The raw data from the IMUs (acceleration and angular velocity) were obtained in the default local coordinate system of the sensors. In order to calculate relative movements, the raw data were rotated into the global coordinate system using quaternions.^27^ Joint torque and power were calculated from the global acceleration and angular velocity vectors.

### Statistical Analysis

The EMG and kinematic variables were examined using repeated measures MANOVA in IBM SPSS version 23 (IBM Corporation, Armond, New York) with Gender and Before/After anaesthesia as between subjects factor, Trials as the within-subjects factor, and a priori significance set at *p* = 0.05. There was no significant within-subjects effect for trials and so the mean data over the three trials was used for any subsequent analyses (for example FFT). Similarly, there was no significant main effect for Before/After anaesthesia nor were there any significant interactions, which indicates that blocking the action of anconeus had little or no effect on the subsequent movement of the elbow joint. However, the analysis did reveal a significant main effect for Gender (Pillai’s Trace 0.657, *F* = 3.517, *p* = 0.034) and subsequent contrasts revealed this was a result of differences in the angular velocity data (*F* = 7.569, *p* = 0.014).

### Finite Element Model

A finite element model of the elbow was developed to investigate the effect on elbow joint contact area and range of motion of blocking of the anconeus. The model was created from a CT scan of the elbow of a healthy 26 year old male.

**Figure 2:**
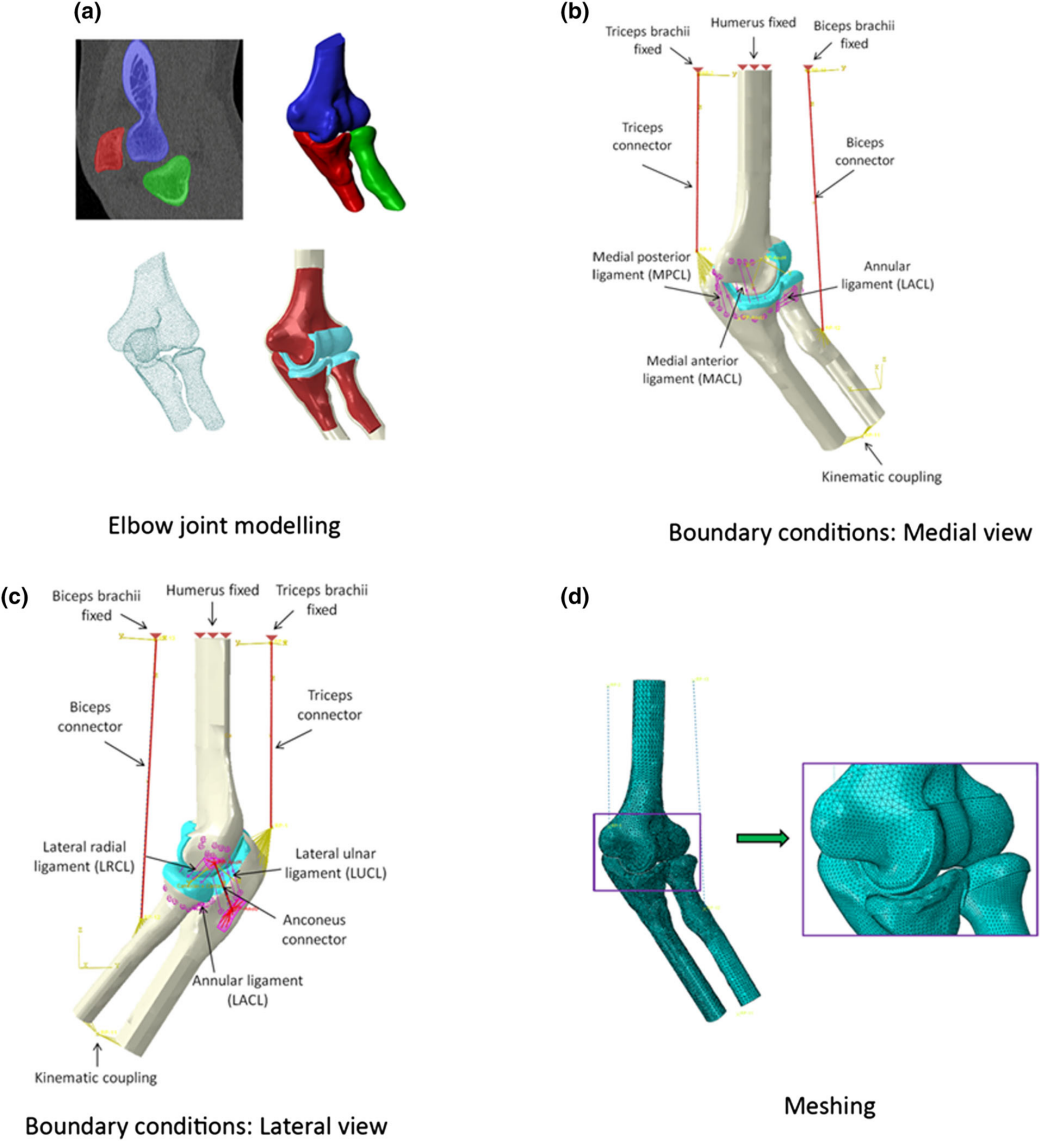
(a) Elbow joint modelling; (b) boundary conditions of the elbow: medial view; (c) boundary conditions: lateral view and (d) mesh of the model.

The 3-D volumetric CT scan data were imported into ScanIP image processing software (ScanIP™ Version 3.2, Simpleware Ltd, Exeter, UK) where the surface geometry of the bones and cartilage was created through a segmentation process, as illustrated in Fig. 2a. The segmented bone and cartilage surface data were exported in point cloud format then imported into SolidWorks (SolidWorks^®^ Dassault Systèmes, SolidWorks Corp, Waltham, MA, USA), enabling solid models to be created from the surface data. The solid models (cortical and trabecular bone and cartilage) were imported into Abaqus CAE (Abaqus/CAE Version 6.12-2, Dassault Systèmes Simula Corp, Providence, RI) where pre-processing tools for solid geometry were employed to produce the final assembled model of the bone and cartilage. The thickness of the cortical bone was approximately two millimetres^43^,^57^ and one millimetre for cartilage. Finally, the ligament and muscle representations were added to the model.

Three-dimensional basic connector elements (CONN3D2, Connector 3D 2-nodes) were employed to represent the muscle behaviour of the anconeus, biceps brachii and triceps brachii muscles. Cartesian and Cardan type connectors were used to simulate the muscle force. The connector elements were positioned at the insertion points of the muscles in positions defined by the literature.^7^,^9^,^40^ The advantage of using connector elements to simulate the concentric contraction of the muscle is that the trochlea notch and the radial head follow the natural path of the flexion–extension motion, so that the contact area between the cartilages could be estimated.

The analysis was initiated with the forearm in the position indicated by the CT scan data, flexed 30° with respect to the longitudinal axis of humerus. Flexion and extension motions were simulated by applying loads to the biceps brachii and triceps brachii connectors respectively to produce a range of motion (RoM) of approximately 90°. The RoM was chosen to correspond with that employed for the flexion–extension experimental trials, enabling the findings of the finite element analysis to be more readily compared with those of the clinical study.

To model the effect of anconeus on the elbow joint, four different loads were applied to the anconeus connector, 0, 9, 18 and 27 N, representing 0, 5, 10 and 15% of the total load applied to the triceps brachii, respectively. The 0 N load corresponded to the case when anconeus was deactivated and 27 N (15%) represented the estimated maximum contribution that anconeus is reported to able to provide to the overall extension force.^56^

### Material Properties

Bone was modelled as an isotropic, homogenous and continuous material. The mechanical properties of cortical and trabecular bone, cartilage and ligaments were taken from the literature.^1^,^2^,^14^,^17^,^19^,^25^,^33^,^47^,^52^

Cortical bone was assumed to have a Young’s modulus of 17.5 GPa and Poisson’s ratio 0.3. The trabecular bone in the model was assigned Young’s modulus and Poisson’s ratio values of 309.8 MPa and 0.3 respectively. Cartilage was modelled as an elastic material with a Young’s modulus of 12 MPa and Poisson’s ratio 0.4.

The collateral ligaments of the elbow were modelled as linear springs using SPRINGA elements in Abaqus CAE. The medial anterior, medial posterior, lateral radial, lateral ulnar and lateral annular collateral ligament representations were assigned stiffnesses of 72.3, 52.2, 15.5, 57.0 and 28.5 N/mm respectively.

### Boundary Conditions

The translation movement and rotation of the humerus in the global x, y and z axes were constrained at the distal border, as shown in Fig. 2b. The origin and insertion of anconeus and the proximal ends of the biceps brachii and triceps brachii connectors were attached to the bone geometry using kinematic couplings (Fig. 2c). The distal ends of the biceps and triceps brachii connectors were fixed in the same way as the humerus. The attachment points of the ligaments in the elbow model were as described and defined in the literature.^4^,^6^,^9^,^11^,^34^,^49^ Four springs were employed to represent each ligament of the elbow, except the annular ligament which was represented with three springs. Finally, the interaction between cartilages was defined as a surface-to-surface interaction without friction. Loads were applied to the muscle connectors to produce a range of motion of 90°. There was no constraining of the rotation of the radius and ulna bones in the model. The radius and ulna were attached using a kinematic coupling in the distal part and in the proximal part they were connected to the humerus using the lateral and medial ligaments. A muscle force was applied to the biceps connector to simulate flexion movement. Then, the biceps brachii connector was deactivated and triceps brachii connector activated to simulate extension motion.

### Mesh Sensitivity Analysis and Validation of the Model

The geometry of the humerus and cartilage of the radius were meshed with 4-node linear tetrahedral elements, C3D4 (Continuum, 3D, 4-node). A mesh sensitivity analysis of the elbow model was undertaken to ensure accuracy of the results. This consisted of keeping the mesh of the humerus bone and cartilage constant then comparing the predicted cartilage contact area obtained under an applied axial force^23^ when different seed sizes were used to mesh the radius cartilage. Global seed size was varied in the range 0.01–0.0003 which generated between 507 and 197,545 model elements for the cartilage. The contact area of the articulation became approximately constant, at around 39 mm^2^, at a global seed size of 0.5 mm (48,380 cartilage elements). Decreasing the seed size further to 0.4 mm resulted in a model with nearly twice as many cartilage elements (91,128), yet predicted cartilage contact area changed by around only 1%. Therefore, a global seed size of 0.5 mm was utilised for cartilage regions in the model and a 2 mm seed size was used for bone (Fig. 2d) as this provided a good balance between accuracy and computation time. The final assembled model consisted of 491,969 4-node linear tetrahedral elements for the bone and cartilage, 22 linear springs for the ligaments, and 3 connector elements for the muscles.

The finite element model of the elbow joint was validated both qualitatively and quantitatively by comparing the contact area pattern with the results from the experimental tests undertaken by Goto *et al*.^21^ and by comparing predicted surface stresses at the proximal ends of the radius and ulna at different flexion angles against those obtained in a cadaveric study.^45^ Goto *et al*.^21^ evaluated the contact area of three healthy elbows *in vivo* using a non-invasive technique and a markerless algorithm. The contact area obtained was found to be in the medial region of the ulna and humerus. The contact area of the radius was found to be in the central region of the head except at 135° where it was in the anterior side of the head.

**Figure 3:**
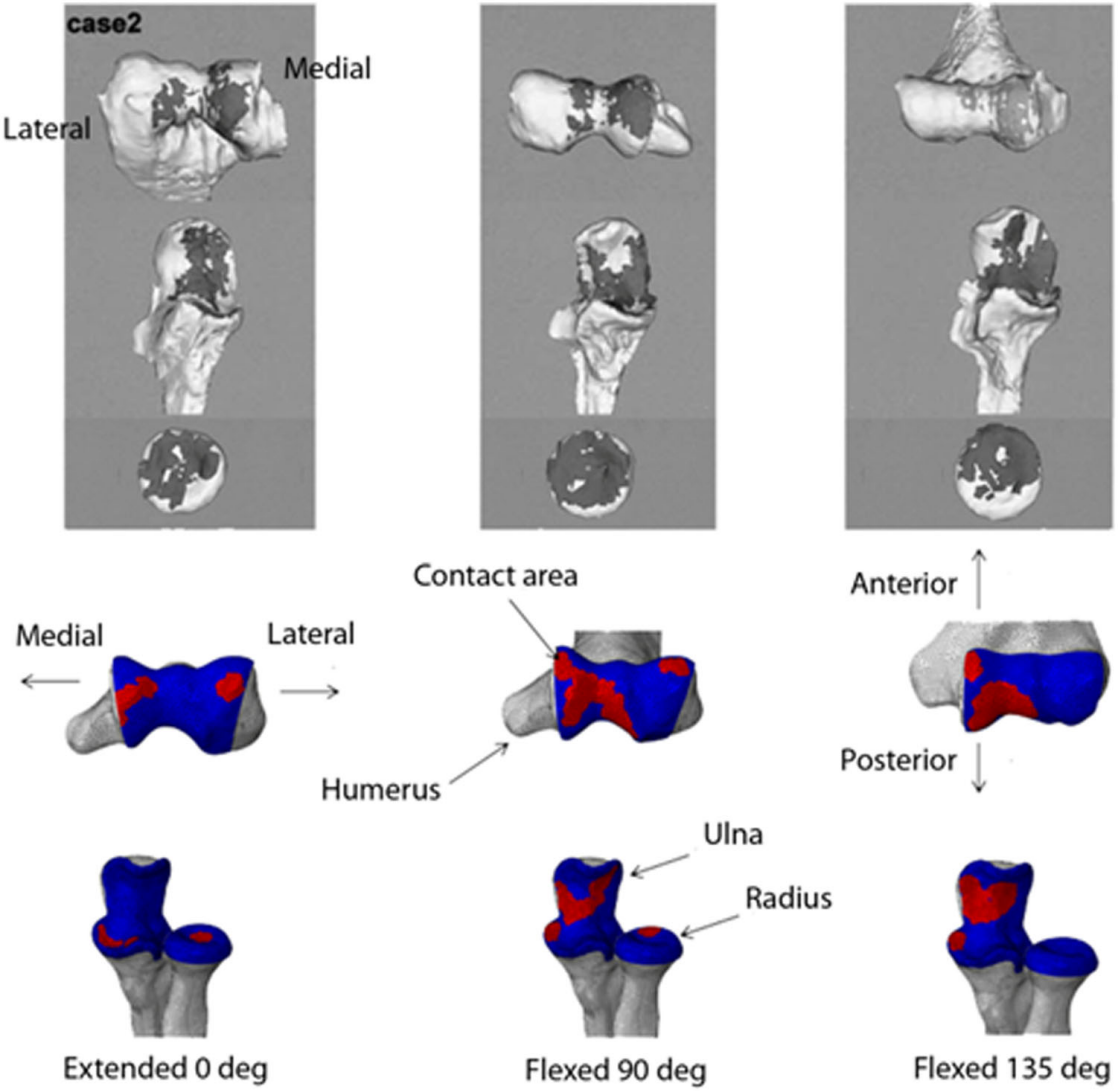
Contact area of the elbow joint at different degrees of flexion: comparison of experimental^21^ (upper portion of the figure) and predicted (lower portion of the figure) results (figure adapted from Goto* et al.* 21).

The contact area pattern produced by the finite element model was in good agreement with the results of the experimental study; in particular, the contact area in the trochlea notch of the ulna appeared on the medial side at the three angles considered and the contact area of the radius at 0° appeared in the centre of the head and moved to the edge at 90° (Fig. 3).

**Figure 4:**
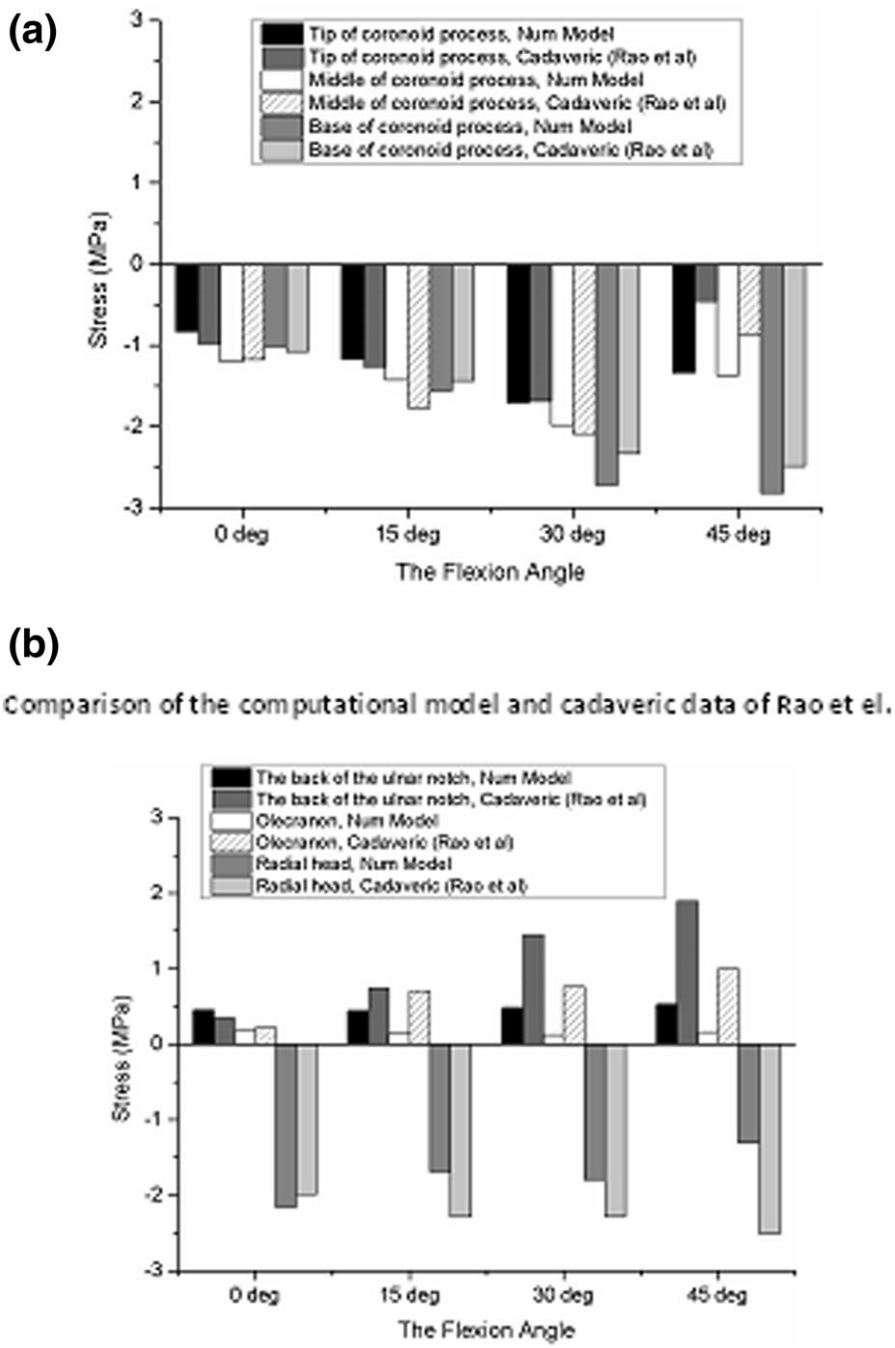
Predicted and measured^45^ surface stresses at six elbow sites and four flexion angles (0°, 15°, 30°, and 45°).

Rao *et al*.^45^ used the strain resistance method to obtain stresses at six sites (tip, middle, and base of the coronoid process; back ulnar notch; olecranon; and radial head) in eight cadaveric elbows at four flexion angles (0°, 15°, 30°, and 45°) after placing the distal ends of the ulna and radius in a neutral position and loading axially in increments up to a maximum of 500 N. Figure 4 shows predicted surface stresses from our model shown alongside corresponding values obtained from the experimental study. It can be seen upon inspection of this figure that predicted stress values and patterns are in good agreement with those obtained experimentally. In particular, at the maximum vertical load, the higher surface stress when the elbow flexion angle was 0° occurred at the middle of the coronoid process, however, as flexion angle increased the higher surface stress shifted to base of the coronoid process.

## Results

### Electrical Activity

#### Anconeus Before and After Blocking

**Figure 5:**
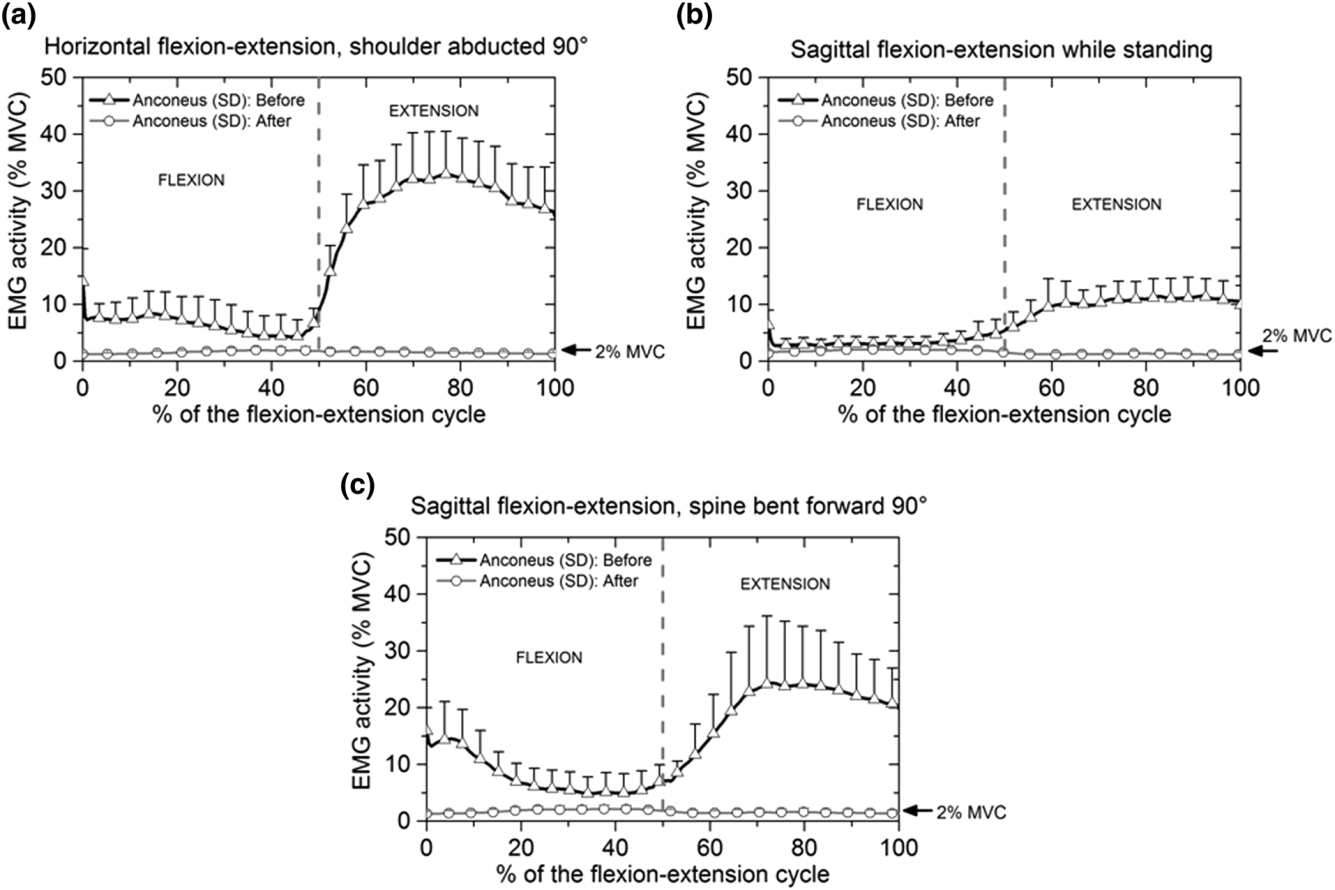
Mean and standard deviation of the relative electrical activity of anconeus muscle before and after applying anaesthesia: (a) flexion–extension cycle with the shoulder abducted 90°; (b) flexion–extension cycle in the sagittal plane while standing and (c) flexion–extension cycle with the spine bent forward 90°. Anconeus activity was greater in extension movements.

The relative electrical activity of anconeus during flexion–extension movements before and after blocking is shown in Fig. 5a–5c. The first half of the cycle (0–50%) represents the elbow flexion and the second half (50–100%) represents elbow extension. The relative electrical activity of anconeus throughout the flexion–extension cycle indicates that anconeus activity before blocking was generally greater in extension than in flexion, Fig. 5a–5c.

Before blocking, during flexion–extension movement in a horizontal plane, the maximum anconeus activity was 8.4 ± 4% MVC in flexion at 15% of the cycle. When extension started anconeus activity was 9.4 ± 3.5% MVC, rising to maximum of 33 ± 7.5% MVC, as shown in Fig. 5a.

In sagittal plane flexion–extension before blocking (Fig. 5b), no anconeus activity was detected during flexion and in extension the electrical activity of the anconeus increased from 5.2 ± 2.2% MVC to 11.4 ± 3.4% MVC between 50 and 82% of the cycle. In sagittal plane flexion–extension with the spine bent forward 90°, the relative electrical activity of anconeus (Fig. 5c) was 14.1 ± 6% MVC at the beginning of flexion. Anconeus activity was 6.9 ± 3% MVC at the start of extension rising to a maximum of 24.2 ± 12% MVC at 72% of the flexion–extension cycle.

In the case of supination–pronation motion with the elbow flexed 90°, no relative electrical activity was detected from the anconeus muscle.

After the anconeus muscle was blocked the relative electrical activity recorded was 2 ± 0.5% MVC during all four movements, a level that corresponds to the value of the background electrical activity obtained from the participants during the static trial prior to the test, consequently anconeus was considered to be inactive.

#### Biceps Brachii Before and After Blocking

**Figure 6:**
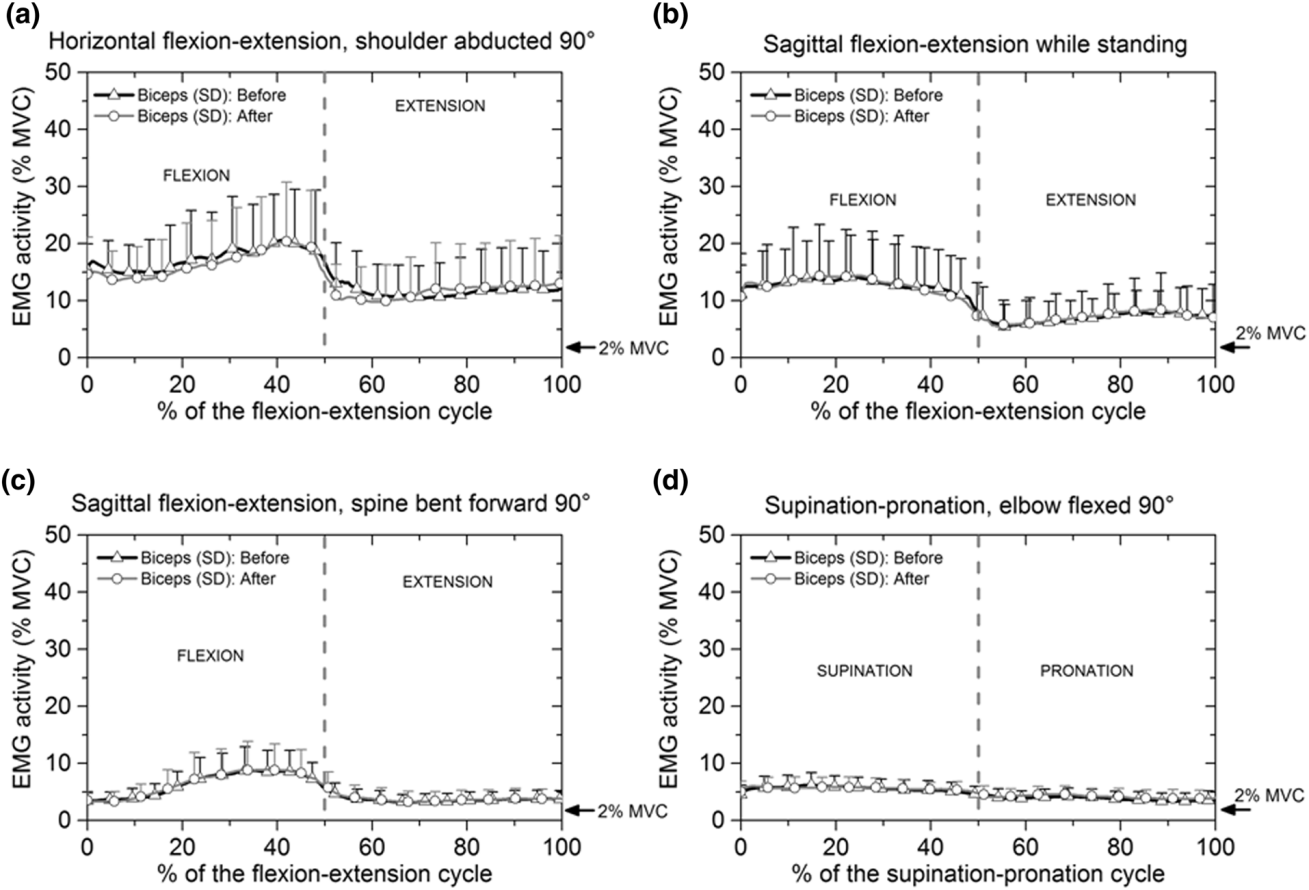
Mean and standard deviation of the relative electrical activity of biceps brachii before and after applying anaesthesia: (a) flexion–extension cycle with the shoulder abducted 90°; (b) flexion–extension cycle in the sagittal plane while standing; (c) flexion–extension cycle with the spine bent forward 90° and (d) supination-pronation with the elbow flexed 90°. Biceps brachii activity remained approximately the same before and after anconeus defunctioning.

The relative electrical activity of the biceps brachii before and after blocking of anconeus is shown in Fig. 6a–6d. The relative electrical activity of the biceps brachii was generally higher in flexion than in extension and slightly higher in supination than in pronation. During flexion–extension in a horizontal plane before anconeus blocking, biceps activity decreased from 19.3 ± 10% MVC to 13.1 ± 7.3% MVC when the flexion motion changed to extension during the cycle (Fig. 6a). In flexion–extension movements with the spine bent forward, the maximum biceps brachii activity recorded was 8.6 ± 4% MVC during flexion at approximately 40% of the cycle, Fig. 6c. During the supination–pronation motion, the relative electrical activity of biceps brachii was slightly higher in supination (6.2 ± 2% MVC) than in pronation (3.5 ± 1.5% MVC), Fig. 6d.

From Fig. 6a–6d it can be seen that the electrical activity of the biceps brachii was similar before and after applying anaesthesia to the anconeus, indicating that blocking of the anconeus had little or no effect on the electrical activity of the biceps brachii for all the movements considered.

#### Triceps Brachii Before and After Blocking

**Figure 7:**
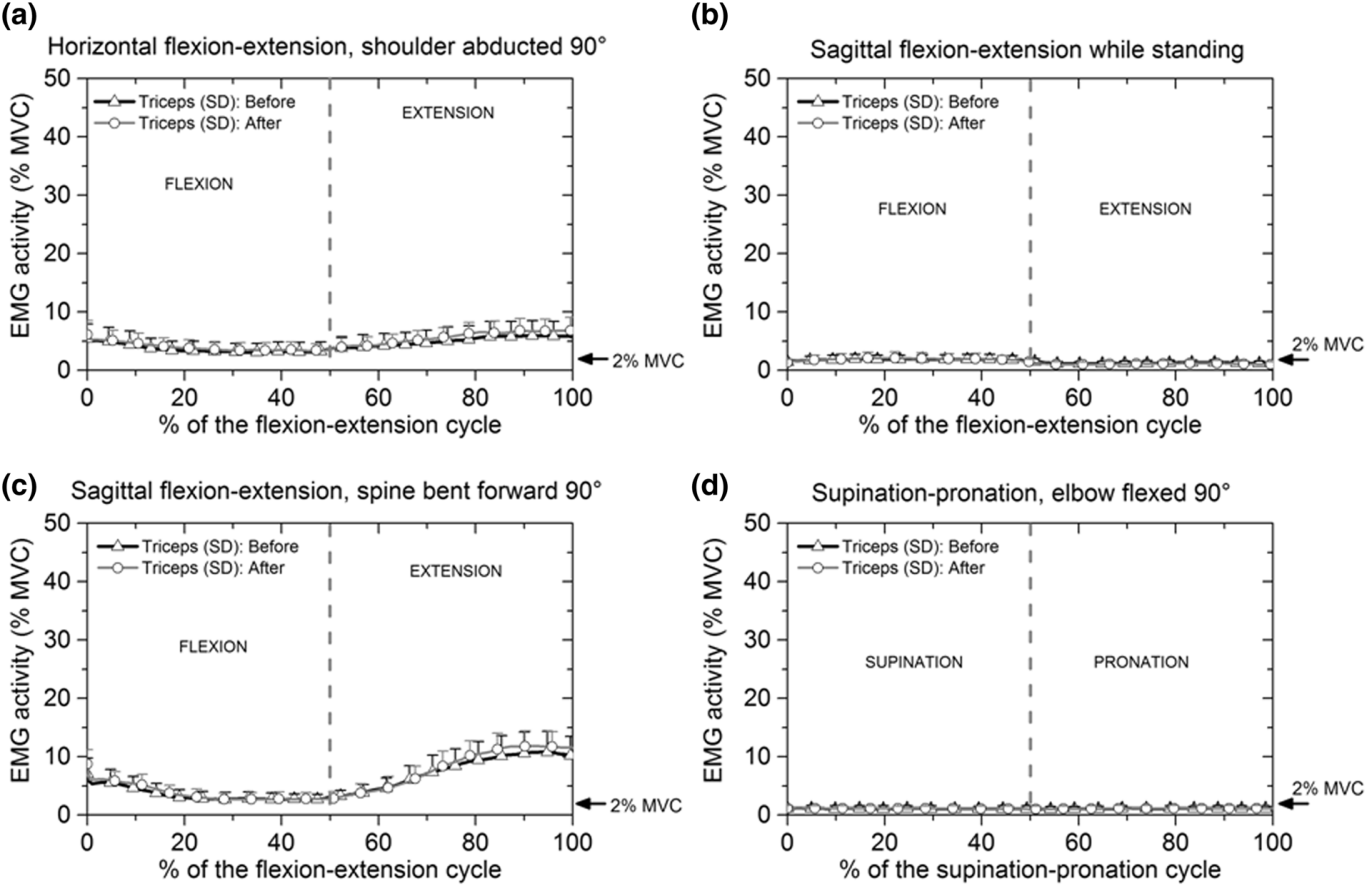
Mean and standard deviation of the relative electrical activity of triceps brachii before and after applying anaesthesia: (a) flexion–extension cycle with the shoulder abducted 90°; (b) flexion–extension cycle in the sagittal plane while standing; (c) flexion–extension cycle with the spine bent forward 90° and (d) supination-pronation with the elbow flexed 90°. Triceps brachii activity was approximately the same before and after blocking anconeus.

Figure 7a–7d shows the relative electrical activity of the triceps brachii before and after blocking of the anconeus. In horizontal flexion–extension before anconeus was blocked, the electrical activity of the triceps brachii gradually decreased during flexion before increasing in extension from 3.6 ± 1.7% MVC to 5.5 ± 2.4% MVC between 50 and 80% of the cycle (Fig. 7a).

In sagittal plane flexion–extension and supination–pronation motions, the relative electrical activity of triceps brachii was at a level at which the muscle could be considered inactive, Figs. 7b and 7d.

In flexion–extension with the spine bent forward, electrical activity of the triceps brachii reduced from 5.7 ± 2.3% MVC at the beginning of flexion to 3 ± 0.8% MVC at the end of flexion. Activity increased during extension, reaching a maximum of 10.6 ± 3.6% MVC at 90% of the flexion–extension cycle as shown in Fig. 7c.

From Fig. 7a–7d it can be seen that blocking of the anconeus did not affect the electrical activity of the triceps brachii.

### Kinematics and Kinetics of the Elbow

#### Kinematics

The elbow angular velocity data before and after anconeus blocking exhibited a strong-linear relationship, with slope in the range 0.98–1 and a Pearson’s correlation coefficient (*r*) of 1 for all cases. The strong-linear relationship between elbow angular velocity before and after anconeus blocking in all four movements indicates that anconeus activity made no significant difference to the angular velocity of the elbow.

A power spectrum analysis^55^ of the relative angular velocity indicated that there was no change in frequency components following application of the anaesthesia to the anconeus, confirming that the angular velocity signal is essentially identical before and after blocking. The main frequency of the angular velocity was approximately the same for all movements, 0.5 Hz.

#### Kinetics

The net torque and power for the elbow joint for the four movements before and after blocking of anconeus suggests that blocking of the anconeus had no effect on the kinetics of the elbow joint in any of the movements.

In addition, total joint work was calculated for each of the four movements both before and after blocking by calculating the area below the power curve in order to confirm that anconeus blocking had no effect on elbow kinetics.

The mean and standard deviation of the total joint work at the elbow for the four movements before and after blocking anconeus did not have a significant effect on elbow joint work.

### Range of Motion of the Elbow Joint

**Figure 8:**
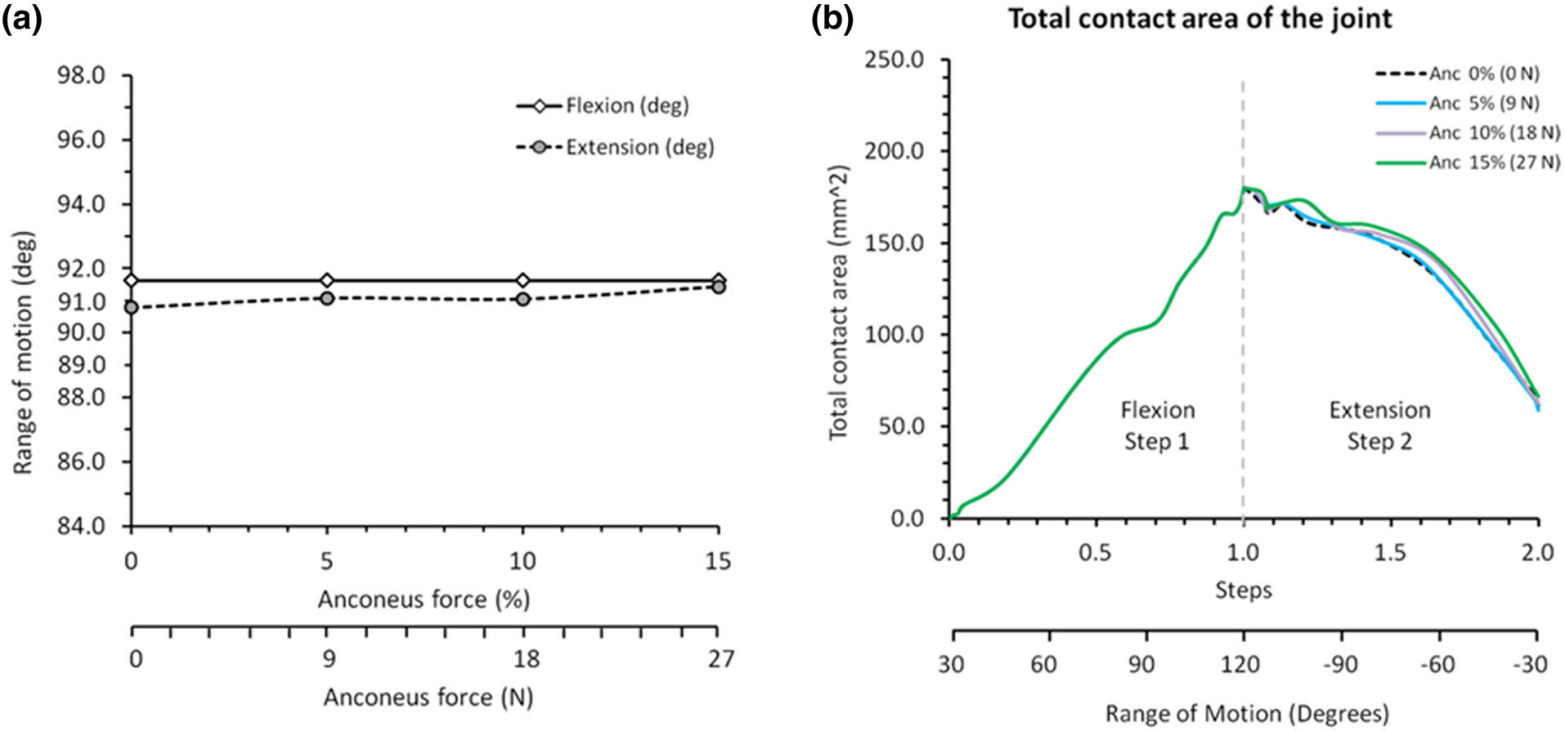
(a) Range of motion and (b) total contact area of the elbow joint.

The range of motion results obtained from the finite element analysis are presented in Fig. 8a, which shows that the range of motion of the elbow in extension was virtually unchanged as anconeus force was reduced. Therefore, it may be inferred that the effect of the anconeus muscle on the range of motion of the elbow joint is not significant when the force applied to the anconeus is less than 15% of the total extension force, an outcome that supports the findings from the experimental trials.

### Contact Area of the Joint

The finite element predictions shown in Fig. 8b demonstrate that reducing the contribution of anconeus has only a minor effect on the elbow joint contact area, again indicating that anconeus contributes little to elbow kinematics.

## Discussion

The results of the current study clearly indicate that the normal relative electrical activity of anconeus before blocking is greater during extension in the flexion–extension cycle,^28^^–^ 30,41,44,53 which, when considered in conjunction with the elbow kinematic data, suggests that anconeus is a weak elbow extensor.

The linear relationship between the elbow angular velocity before and after anconeus deactivation suggests that the anconeus contribution to the elbow kinematics is not significant. Furthermore, the spectrum frequency analysis of the angular velocity before and after anconeus blocking indicates that the frequency components do not change. The anconeus muscle and triceps brachii were inactive during unresisted pronation–supination movement, a finding consistent with previous studies,^41^,^53^ implying these muscles do not contribute to forearm rotation.

The electrical activity of the biceps brachii recorded indicates that the muscle contributes more in the flexion motion than extension whereas the triceps brachii contribute more in extension. Blocking of anconeus did not have a significant effect on the relative electrical activity of the biceps and triceps brachii muscles. In addition, the net torque, power and work at the elbow before and after anconeus blocking remained the same, which indicated that anconeus does not significantly contribute to the elbow kinetics.

This investigation considered a relatively limited number of movements; however, the selected movements were considered as representative of those common in daily life activities. Furthermore, the movements were chosen so that the effect of the gravitational toque at the elbow joint could be used to clarify changes in muscle activity in kinematics.

In the finite element analysis, the range of motion and contact area of the elbow model did not change significantly after blocking of the anconeus was simulated, supporting the findings of the experimental trials suggesting the anconeus does not contribute significantly to elbow joint kinematics.

As is the case with all numerical analyses of this type, the finite element model is subject to limitations and simplifications. For example, linear representations were used to describe behaviour of the soft tissues (ligaments and cartilage) in the model. Ligaments are known to exhibit non-linear viscoelastic behaviour. A consequence of this is that if ligament strain was sufficiently low that behaviour was in the non-linear region, then the model would predict less joint motion than would occur in reality due to the overestimation of ligament stiffness. However, it has been reported that ligaments tend to operate at the end of the toe-region, close to the linear region, in which case the error induced from a linear model would not be significant.^52^ Moreover, the model was concerned with the final elbow position following application of the load, therefore at this point viscoelastic effects would be reduced.^52^ Also, accurate representation of ligament behaviour generally requires a large number of parameters to be specified for which accurate data are typically not available.^54^ A recent sensitivity study undertaken by Kim *et al.* 26 found that a change of ±10% in ligament stiffness made essentially no difference to joint contact stress and contact area in their finite element elbow joint model, the predictions from which compared favourably to measured values obtained using pressure sensitive film on cadaveric elbows subjected to axial loads. In addition, accurate predictions were obtained without the use of ligament pre-stress for which available data are limited.^54^

Cartilage in our model was also assumed to behave elastically although it is recognised that a neo-Hookean hyperelastic model would generally provide a more accurate representation of its behaviour. In the study undertaken by Kim *et al.* 26 the authors studied the effect of treating elbow joint cartilage as a linear elastic compared to a neo-Hookean hyperelastic material. They found little difference in contact area and stress between the predictions obtained when the two different material behaviour representations were employed.

Furthermore, our model was validated by comparing predictions with elbow joint contact area patterns and surface stresses at the proximal ends of the radius and ulna at different flexion angles obtained from two cadaveric studies, suggesting that the material properties employed in our model are sufficiently accurate for this research.

In conclusion, the results of this study suggest that anconeus is a weak extensor of the elbow and that, following blocking, the relatively small contribution of the anconeus to extension before blocking is compensated by the triceps brachii. This supports the view that the anconeus is essentially an accessory muscle and so its sacrifice in clinical applications would not significantly affect the kinematics and kinetics of the elbow.^18^,^31^,^37^,^50^
